# Mycotoxins Detection and Fungal Contamination in Black and Green Tea by HPLC-Based Method

**DOI:** 10.1155/2020/2456210

**Published:** 2020-08-03

**Authors:** K. Pakshir, Z. Mirshekari, H. Nouraei, Z. Zareshahrabadi, K. Zomorodian, H. Khodadadi, A. Hadaegh

**Affiliations:** ^1^Department of Parasitology and Mycology, Basic Sciences in Infectious Diseases Research Center, School of Medicine, Shiraz University of Medical Sciences, 7134845794 Shiraz, Iran; ^2^Department of Parasitology and Mycology, School of Medicine, Shiraz University of Medical Sciences, 7134845794 Shiraz, Iran

## Abstract

The fungal contamination and total aflatoxins (AF) and ochratoxin A (OTA) of tea samples were examined. A total of 60 tea samples were extracted and treated with immunoaffinity columns. The amount of AF and OTA were determined by using high-performance liquid chromatography (HPLC) with a fluorescence detector (FD). Tea samples were cultured and the fungi were identified. The results showed that 24 (40%) samples were contaminated with AFs and none of the tea samples were above the acceptable limit of AFs (≥10 *μ*g/kg). All of the samples were contaminated with OTA where only 3 black tea samples (6.6%) and 1 green tea sample (6.7%) were detected to have more than the standard limits of toxin (10 *μ*g·kg^−1^). The mean concentration of OTA in the black tea was higher than green tea. *Aspergillus niger* was the predominant fungi isolated from black and green tea samples. Considering the high contamination of mycotoxins in tea samples, regular monitoring in the tea process for improving quality is recommended.

## 1. Introduction

Tea (*Camellia sinensis*) is one of the most common aromatic beverages around the world. It is an enjoyable and widely consumed drink after water [1]. There are five basic types of tea with different tastes which are Black, Green, Oolong, Pu-erh, and White. All types of tea have a strong aromatic flavor and contain theine and caffeine, but less than coffee [2]. Also, tea has been used as a traditional treatment for different conditions, such as blood pressure reduction, decreasing LDL cholesterol, being antidiabetic, recovering gut health, improving heart health, and reducing the risk of stroke and cancer. Black tea has many antioxidants compounds that are health beneficial and it can also help to reduce inflammation. Furthermore, green tea is one of the healthiest beverages on the planet and it is rich in polyphenol antioxidants, including catechin and minerals [3]. These combinations can have influential effects on health, including improvement of brain function and exhibits antineurodegenerative (anti-Parkinson and anti-Alzheimer) and antidepressant effects. These substances can also reduce the formation of free radicals in the body which have an important role in aging and precursing many forms of diseases. Tea also contains many dietary components, including vitamin E, C, fluoride, and potassium [4, 5]. Mycotoxins are secondary fungal metabolites that produced by specific strains of mold (fungi) which are famous as potential top priority human's health hazard. Mycotoxins may cause minor to severe ailment including skin necrosis, leukopenia, immunodeficiency, and even liver cancer [6]. Due to the nonprotein structure of these toxins, they are often resistant to heat and might compromise the health of those consuming such contaminated foods, despite the cooking process [7]. Among the several various mycotoxins that have been identified, aflatoxins (AFs) and ochratoxin A (OTA) are the most commonly observed toxins that contaminate different agricultural commodities, including tea, coffee, nuts, and spices [8]. Aflatoxin is a mycotoxin produced by some members of the *Aspergillus* section flavi including *A*. *flavus*, *A*. *parasiticus*, and *A*. *nomius* and has a poisonous effect on the liver [9]. Aflatoxins are fluorescent heterocyclic secondary metabolites with molecular weights of 286 to 346 Dalton. The major AFs classified as B1, B2, G1, and G2 are particularly hazardous to humans and animals, which can be identified based on their fluorescence under blue or green light and their relative mobility during separation by thin layer chromatography (TLC) [10]. In this family, AFB1 is a well-known toxin with mutagenic and carcinogenic properties for both humans and animals [11]. Aflatoxin contamination in tea is a serious worldwide concern affecting international trade. The acceptable concentration of this toxin varies from 5 *μ*g·kg^−1^ for AFB1 to 10 *μ*g·kg^−1^ for total AFs [12]. Ochratoxin is another mycotoxin that has a poisonous effect on the kidney and is primarily produced by *Aspergillus ochraceus* and *Penicillium verrucosum*. The acceptable concentration is 10 *μ*g·kg^−1^ for OTA [12, 13]. Previous studies have reported that teas support fungal growth and consequent mycotoxin production. Carraturo et al. reported that *Aspergillus niger* and *A*. *tubingensis* were detected from 70% of the samples. Fifty percent of those samples were contaminated above the acceptable limit of mycotoxin for food products [14]. Sedova et al. reported that average contamination level corresponds to the exposure of 3–40% (aflatoxin B1) and 5–24% (ochratoxin A) of mean overall estimates for different cluster diets [15]. As most of the teas in the Iranian markets are imported, and there is less information concerning fungal contamination and mycotoxins level on black and green teas, this study is designed to evaluate the load of fungal contamination and determine the concentration of aflatoxins and ochratoxin A in black and green teas in Shiraz, southern Iran.

## 2. Materials and Methods

### 2.1. Sampling

A total of 60 tea samples, commercially and unpacked, were randomly purchased from supermarkets and the attorney's market of Shiraz. The samples include 45 black teas and 15 green teas from different brands.

### 2.2. Fungi Screening and Load of Contamination

Five grams of each tea sample was added to 45 ml of sterile distilled water containing 0.05% tween 80, shaken with the vortex for 2 min, then 100 *μ*l of suspension was inoculated on to sabouraud dextrose agar containing chloramphenicol and 6% NaCl (Sigma Chemicals, USA) and incubated at 25°C till 7 days. The fungal colonies were counted, and the total fungal load was calculated and reported as a colony-forming unit (CFU) per gram based on International Commission on Microbiological Safety for Foods (ICSMF) protocol. The colonies were subcultured to determine the qualitative/quantitative outcome and identified as the taxonomic schemes for *Aspergillus*, *Penicillium*, *Mocur*, and other unknown fungi [16–18].

### 2.3. Mycotoxin Determination

#### 2.3.1. Aflatoxins

A stock solution of AFB1 and AFG1 (1000 *μ*g·kg^−1^), and AFB2 and AFG2 (200 *μ*g·kg^−1^) was dissolved in benzene-acetonitrile (98 : 2, v/v), purchased from Supelco (Bellefonte, PA, USA). The working standard solution (500 *μ*g·kg^−1^) for each mycotoxin was prepared, kept in an amber vial, and stored at −20°C until use. The AFs' standard calibration curves for HPLC determination were prepared by dissolving an appropriate amount of working standard solution in the same solvent to obtain the final concentration. The glass microfiber filter was obtained from BOECO, Germany (15 cm, 934-AH), and fluted filter paper (24 cm), methanol, acetonitrile (HPLC grade), and sodium chloride were purchased from Merck Darmstadt, Germany. Immunoaffinity columns for AFs were obtained from Ibios Pontcharra-Sur-Turdine, France; the decontamination of the glassware was performed using a sodium hypochlorite solution and it was washed to a neutral pH with distilled water [19].

#### 2.3.2. Ochratoxin A

HPLC-grade acetonitrile, acetic acid glacial, and sodium chloride were purchased from Merck Darmstadt, Germany, and the OTA (1000 *μ*g·kg^−1^) was obtained from Supelco Bellefonte, USA. A standard working solution of OTA (100 *μ*g·kg^−1^) was prepared by evaporating a known volume of the stock solution under a nitrogen stream, followed by dissolving it in methanol-water (50 : 50, v/v). The immunoaffinity columns for the OTA to lead ochratoxin A (OTA) contained extract to the antibody; the glass microfiber filter (BOECO) (15 cm, 934-AH) and the fluted filter paper (24 cm) were obtained from Germany. Phosphate-buffered saline (PBS) was prepared by adding the following chemicals to one liter of water: anhydrous dibasic sodium phosphate (2.04 g), sodium chloride (87.9 g), and sodium dihydrogen phosphate monohydrate (12.62 g). One hundred milliliters of this solution was diluted and the pH was adjusted to 7.4 with sodium hydroxide. The normal distribution of data was verified using the Kolmogorov–Smirov test. HPLC technique chromatographic separations were performed on a HPLC system, Agilent 150 Technologies SL 1200 Series (Waldbronn, Germany) composed of a binary pump equipped with microvacuum degasser, thermostated autosampler, column compartment, and fluorescence 152 detectors (model G1321 A). Online photochemical derivatization was performed using a commercially available system UVETM LCTech GmbH (Dorfen, Germany) placed between the 154 separation column and the fluorescence detector, which consisted of a 254 nm low-pressure Mercury lamp and a 1 mL knitted reaction coil, fitted around the UV lamp. All the 156 separations were performed by using a ZORBAX Eclipse XDB C18 column (150 × 4.6 mm i.d., particle size 5 *μ*m, Agilent Technologies) operating at a flow rate of 1.0 mL/min in isocratic elution with a 158 mixture of water, methanol, and acetonitrile (55 : 15 : 30, v/v/v). The injection volume was 20 *μ*L, and the column temperature was set at 40°C. The fluorescence detection was carried out at a *λ*exc160 and *λ*em of 365 and 435 nm, respectively. The system was interfaced, via network chromatographic software (Agilent ChemStation), to a personal computer for data acquisition and processing.

### 2.4. Standards and Reagents

All chemicals except methanol and acetonitrile were laboratory-grade mixtures, acquired from Merck, Germany. Standards including AFB1, AFB2, AFG1, AFG2, and OTA were purchased from Supelco (Bellefonte, USA).

### 2.5. Sample Treatment and Purification

1 gr of NaCl and 20 cc methanol (80%) were added to 5 grams of the grinded tea samples and shaked for 3 minutes and then filtered. Formerly 65 cc of distilled water to the solution was added and finally the resulting solution from the filter through this filtered solution was collected. For purification of AFs from tea, 70 ml of the filtrated solution was passed through the IAC Afla column (Libios) at a flow rate of 1 ml·min^−1^. Then, 10 ml of sterilized, deionized water was passed through the column at a flow rate of 1 ml·min^−1^. The AFs were subsequently eluted from the column, with 1.5 ml of methanol and collected in a glass vial; then 1.5 ml of sterilized, deionized water was added. 100 *μ*l of elute was injected into the HPLC. For purification of OTA from tea, 50 ml of the filtrated solution was passed through the IAC column (Libios) at a flow rate of 1 ml·min^−1^. The OTA was subsequently eluted from the column, with 1.5 ml of methanol-acetic acid (98% methanol, 2% acetic acid), and collected in a glass vial; then, 1.5 ml of sterile, deionized water was added. Finally, 100 *μ*l of the elute was injected into the HPLC [19].

### 2.6. Validation and Quality Assurance Method

For AFs linearity, a seven-point calibration curve was built with concentrations of 0.1, 0.5, 1, 2, 5, 10, and 20 *μ*g·kg^−1^ for AFB1, AFB2, AFG1, and AFG2. The calibration curves were obtained using the linear least squares regression procedure of the peak area versus the concentration. The limits of detection (LOD) and the limits of quantification (LOQ) were determined using the signal-to-noise approach, defined at that level, resulting in a signal-to-noise ratio of approximately 3 : 1 and 10 : 1, respectively. Regarding the accuracy of the method and due to the absence of certified reference material (CRM) available, 25 g of an aflatoxin-free sample of each tea was spiked with AFB1, AFB2, AFG1, and AFG2 at levels of 3, 5, and 10 *μ*g·kg^−1^. The spiked samples were analyzed by the HPLC, as described above, and then the recovery and standard deviation (SD) were calculated. All tests were carried out in three replicates. For the OTA, a five-point calibration curve was built with concentrations of 0.5, 2, 5, 10, and 30 *μ*g·kg^−1^. A calibration curve was obtained using the linear least squares regression procedure of the peak area versus the concentration. The limits of detection (LOD) and of quantification (LOQ) were determined using the signal-to-noise approach, defined as per the concentration, resulting in a signal-to-noise ratio of approximately 3 : 1 and 10 : 1 for LOD and LOQ, respectively. Due to the accuracy of the method applied, and since there was no certified reference material (CRM) available, 25 g of different OTA-free tea samples was spiked with OTA at the levels of 1, 5, and 20 *μ*g·kg^−1^. All the tests were carried out in three replicates, and then the recovery and standard deviation (SD) were calculated [19, 20].

### 2.7. Quality Control

**Figure 1:**
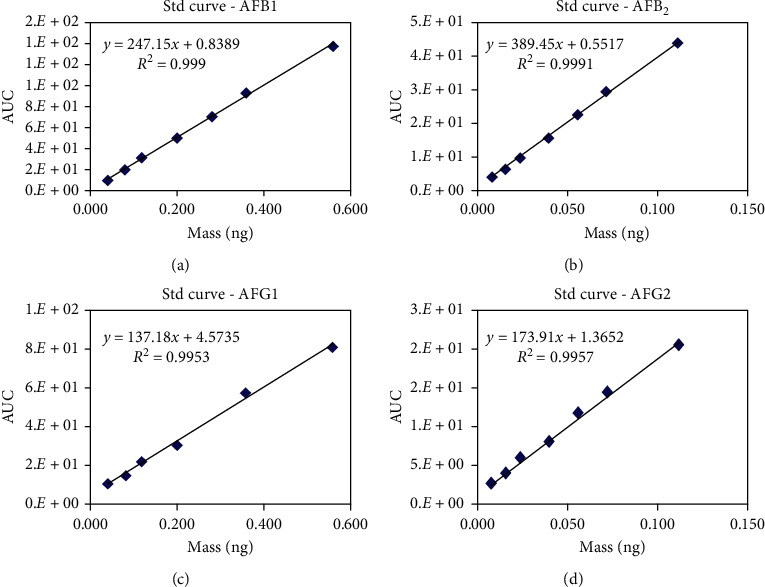
Standard curve of AFB1 (1), AFB2 (2), AFG1 (3), AFG2 (4).

Furthermore, by using confirmed methods, internal and external quality control trials were conducted. Regarding the internal quality control, the precision and exactness of the methods were confirmed. For this purpose, AFB1, AFB2, AFG1, AFG2, and OTA retrievals were recorded by analyzing a blank sample, spiked at 5 *μ*g·kg^−1^ for AFB1 and AFG1, 1 *μ*g·kg^−1^ for AFB2 and AFG2, and 5 *μ*g·kg^−1^ for OTA. The recovery rate for AFB1 was 85%–90% and the usual coefficient of variation was 1.4%. Moreover, the recovery rate for OTA was 93 *μ*g·kg^−1^. The AFs and OTA levels were corrected, according to the recovery value. The LOD and LOQ for AFB1 were 0.33 *μ*g·kg^−1^ and 1 *μ*g·kg^−1^, respectively, and for OTA, it was 1 *μ*g·kg^−1^. The measurement of determination (*R*^2^) was used to calculate method linearity. A sequence of working standard solutions for AFB1, AFB2, AFG1, and AFG2 was prepared. Calibration curves were created by distinct conspiracy area against concentration. *R*^2^ values were considered using the regression equations (Figure 1).

As shown in Table 1, the validation of the HPLC method was carried out in terms of linearity, the LOD and LOQ, accuracy and precision (RME and RSD), and recovery studies.

### 2.8. Toxin Analysis

The detection of AFs and OTA was carried out using an HPLC apparatus (Agilent 1100 series, USA). Liquid chromatography separation was performed on a reversed-phase C18 (25 cm × 0.46 mm × 5 *μ*m), Zorbax eclipsed XDB column, from Agilent (California, USA). The fluorescence detector was set to an excitation wavelength of 365 nm and an emission wavelength of 435 nm. The mobile phase consisted of a combination of acetonitrile-methanol-water (2 : 3 : 5, v/v). It was filtered using a Millipore filtration apparatus, maintained at a flow rate of 1 ml/min^−1^. An electrochemical cell (model Libios-K01, France) was used as postcolumn derivatization. Aflatoxins and OTA were identified by their constant retention times. Calibration curves of peak areas versus AFs and OTA concentrations were then plotted and used for their determination of samples. A waring blender and a water purifier were utilized [20].

### 2.9. Statistical Analysis

The descriptive statistics (mean, standard deviation, range) were calculated by the SPSS software (SPSS Institute Inc., 2000, Version 10.0). The chi-square test was used for the determination of the statistical significance of means differences. A probability value < 0.05 was used to determine the statistical significance.

## 3. Result

All the AFs (B1, B2, G1, and G2) were well separated from each other in the standard and sample chromatogram. AFG2 was elected first, followed by AFG1, AFB2, and AFB1, respectively. The linearity in the working standard solutions at three determinations of five concentration levels was reliable between 0.9999 for AFB1, 0.9991 for AFG2, and 0.9968 for OTA (Table 1). The linearity of the working standard solutions at two determinations of five concentration levels was reliable (0.9997), as exhibited by the coefficient of the determination, i.e., *R* squared (*R*^2^).

The quantity of AFs and OTA in the teas (black and green) is illustrated in Table 2. Among the 60 tested samples, 20 (33.3%) were contaminated with AFs (range: 0.67–2.81 *μ*g·kg^−1^). Of the 60 samples, none of the tea samples were above the acceptable limit AFs (≥10 *μ*g·kg^−1^). The mean concentration of aflatoxin in the black and green tea samples was 1.45 *μ*g·kg^−1^ and 1.93 *μ*g·kg^−1,^ respectively. Out of the 60 analyzed tea samples, all of them were contaminated with OTA, of which only 3 black tea (6.6%) and 1 green tea (6.7%) samples were detected to have more than the standard limits of toxin (10 *μ*g·kg^−1^). The mean concentration of this toxin was higher in the black tea samples. There was a significant difference in the amount of AFT and OTA between the two types of teas (*p* value: AFT = 0.006 and *p* value: OTA = 0.001).

Concentrations of B1, B2, G1, and G2 aflatoxins in green and black tea samples are shown in Table 3. All of the samples have been contaminated less than the detection limit value of the analytical methods.

Various genuses of molds and yeast were isolated from tea samples. Out of 45 samples of black tea, 40 (89%) had fungal contamination. *Aspergillus* spp. (69%) and yeast (6.7%) were the highest and lowest of fungi genus detected, respectively. All of the green tea samples had fungal contamination. The most fungal isolated was yeast (67%) and the lowest rate was *Fusarium* and black fungi (13%) (Table 4).

### 3.1. Load of Fungal Spore

The highest and lowest loads of mold contamination in black tea samples were 30 cfu/gr (*A*. *niger*) and 1560 cfu/gr (*Penicillium*), respectively. The highest and lowest loads of fungal contamination in green tea samples were 60 cfu/gr (*Penicillium*) and 1560 cfu/gr (*A*. *niger*), respectively, in which all spore levels were below the standard level of food safety (10000 cfu/gr-ICSMF).

In the positive cultured samples, all were in acceptable aflatoxin level. Among samples with positive fungal culture, only two samples were contaminated with more than the allowed OTA level, whereas 1 sample was contaminated with an overlimited level of OTA culture and yielded a negative result (Table 5). There was no significant difference between the amount of OTA and load of fungal spore (*p* value: 1.0).

## 4. Discussion

Nowadays, mycotoxins in foodstuffs have become a major problem around the world. Most of the foods and beverages are contaminated with different mycotoxins during preparation, packaging, and transporting before getting consumed, and unfortunately, it covers a wide range of foodstuffs. Diella et al. analyzed 124 samples of different types of nuts. Twenty samples of these foodstuffs (16.1%) were contaminated with AFs. These results showed that among the different types of nuts, pistachios were more susceptible to this mycotoxin. The major of contaminated products with AFs came from Asia. Specifically, samples came from Iran (5/19; 26.3%), Afghanistan (4/19; 21.1%), Uzbekistan (4/19; 21.1%), Tajikistan (2/19; 10.5%), Turkey (2/19; 10.5%), China, and Georgia (1/19; 5.3%) [21]. It is noteworthy that Montagna et al. reported the state of the art of Italian legislation, in the European context, concerning the levels of important mycotoxins in foodstuffs such as maximum levels of AF and OTA in specific food; in addition, legislation for sampling and official controls of mycotoxin was surveyed [22]. However, Montagna et al. examined the presence of AFM1 in 265 cheese samples at different steps of ripening. In this study, AFM1 was found in 16.6% of the cheese samples of which the highest positive incidence was for medium and long-term cheeses during ripening [23]. Currently, drinking tea became the most common beverage among the Britons, who started large-scale production and commercialization of the plant in India during the 17th century. While coffee is known as the drink of choice in most of the Western world, tea dominates in the Eastern part of the world (and the UK) [24]. Tea is one of the most popular drinks in the world, especially in Iran. Considering the high percentage of consumed tea in Iran, most of the tea in the Persian market is imported from abroad and might be contaminated with fungi that potentially produce toxins during pre- or postharvest, storage, processing, and packing stages [25]. Mycotoxins are unavoidable contaminants in foodstuffs worldwide. These toxic chemicals represent a big challenge for food safety and also develop massive country economic damage to the agriculture organization [26]. In comparison to other agricultural products, tea can be very susceptible to mycotoxin contamination. There are two possible causes of mycotoxins contamination in tea: drought stress in winter (preharvest) and undesirable moisture and temperature conditions during storage (postharvest) [27]. The concentration of mycotoxins can be measured by various methods such as ELISA and HPLC. In this study, the HPLC method was used to measure mycotoxin, which has high sensitivity and specificity in comparison with the ELISA method [28]. The amount of aflatoxins consumed contributes to the mutagenic, carcinogenic, teratogenic, and immunosuppressive health effects causing chronic diseases such as liver cancer, haemorrhage, edema, and even immediate death [29, 30]. Aflatoxin B1 is one of the most important among mycotoxins that if taken 10 *μ*g/kg daily for a long period of time can cause temporary adverse effects and by taking 50 *μ*g·kg^−1^ daily it can be the reason of clinically significant effects [30, 31]. The acceptable range of aflatoxin contamination in different countries is varied. The allowed total of aflatoxins in foodstuffs in the United States is 20 *μ*g·kg^−1^ and 2–4 *μ*g·kg^−1^ in Europe. In Iran, this level is 2 *μ*g·kg^−1^ for food and 10 *μ*g·kg^−1^ for tea [29]. Although in our study the aflatoxin concentration level was less than 10 *μ*g·kg^−1^, with long-term consuming it causes irreversible effects. Ochratoxin A is a potent toxin that causes kidney damage in animals [32, 33]. The result of the in vivo study in pig revealed that ochratoxin A has the ability to cause mycotoxic nephropathy and expressed significant effect in proximal tubules in kidneys [34]. In the present study, 100% of samples were contaminated with ochratoxin A (0.5 to 30.51 *μ*g·kg^−1^). Teman's et al. in Turkey reported the amount of ochratoxin A in 50 samples of black tea, in which 4 samples were above the limit level [34] (OTA = 19.6–56.7 *μ*g·kg^−1^). However, in our study, two samples of black tea were contaminated above the limit level of OTA (14/25 and 30/51 *μ*g·kg^−1^). Mogensen et al. did not detect any ochratoxin A from black tea samples [35]. In our study, the difference between the amount of total aflatoxin and ochratoxin A in black and green tea samples was significant statistically, which means that the quantity of toxins in black tea is higher than green tea. According to this result, fermentation process in black tea has no effect in decreasing mycotoxin in comparison with green tea samples [36]. Genus of *Aspergillus* could produce toxin under special conditions. *Aspergillus flavus* required the optimal temperature and time for producing toxin in average 25°C in 7 to 9 days [29]. Comparison of fungal growth and mycotoxin production showed that in some samples, despite the fungal growth, mycotoxin was not detected and vice versa. Considering the above-mentioned conditions, in samples with less fungal contamination but high toxin, mycotoxin was produced during processing. Also, some kind of *Aspergillus flavus* species are belongs to nontoxinogenic group and could not produce any mycotoxin after growing. On the other hand, if the conditions are unfavorable the fungi may be eliminated but the produced toxins will mostly remain. Ochratoxin A could be produced by two main genuses of fungi, *Aspergillus* and *Penicillium* [13]. In this study, 3 samples had higher levels of ochratoxin A (10 *μ*g·kg^−1^). *Aspergillus niger* was isolated from two samples, but one samples had no fungus. It can be assumed that ochratoxin might be produced before final products. In our study, different varieties of hyaline, dematiaceous, and yeasts were isolated from samples. Kazemi et al. showed that 73% of 100 samples had fungal contamination with *Aspergillus* and *Penicillium* [37]; that data was semisimilar to our study. *Aspergillus acidus* and *Aspergillus niger* from 47 samples have been isolated by Mogensen et al. in Denmark [35]. Storari et al. isolated *Aspergillus ovori*, *Aspergillus acidus*, *Aspergillus niger*, and *Aspergillus tubingensis* from 16/22 samples of black tea [38]. We concluded that the diversity of the fungal genus in our study was higher than in those studies.

## 5. Conclusion

Consumption of black and green tea is common among Iranian people, and the mycotoxin is highly resistant to temperature, so care must be taken in quality control of tea production. Although, the standard level of toxicity in different countries is different and current studies have shown a normal range of aflatoxin levels in tea samples. But this level of toxicity will rise by continuous daily consumption of tea because of the accumulation capability of this toxin in the human body. Furthermore, considering that mycotoxins are also present in other food products through daily diet might also elevate mycotoxin level in the body. As a final point, tea is mostly imported in our country. Cooperative and continuous efforts of governmental control authorities and academia are required for the prevention of toxigenic fungi and mycotoxin production and to advance such detection techniques in order to improve food safety.

## Figures and Tables

**Table 1:** Validation parameters for the HPLC determination of AFB1, AFB2, AFG1, AFG2, and OTA.

Validation parameters	AFB_1_	AFB_2_	AFG_1_	AFG_2_	Total AFs	OTA
LOD (*μ*g·kg^−1^)	0.1	0.2	0.3	0.2	0.6	0.47
LOQ (*μ*g·kg^−1^)	0.4	0.7	0.9	0.6	1.8	1.23
Correlation coefficient (*R*^2^)	0.9989	0.9991	0.9953	0.9995	0.09978	0.9968
Recovery (%)	82.5–84.3	74.1–83.1	77.7–88.8	75.0–75.9	74.1–83.1	83.7–99.6
RSD (%, *n* = 20)	1.31	3.77	2.35	2.81	1.77	1.64
RME (%, *n* = 20)	-3.4	-1.0	1.8	-2.1	1.6	2.6

**Table 2:** Concentration of mycotoxin in tea samples.

AFT		OTA	
Samples	No. of positive samples	Mean (range) ± SD	No. of positive samples	Mean (range) ± SD
Black tea (45)	18	1.45 (0.67–4.2) ± 1.09	41	6.09 (5.08–30.86) ±1.27
Green tea (15)	2	1.9 (1.0–2.81) ±1.23	11	2.81 (0.54–20.35) ± 5.95

**Table 3:** Types of aflatoxins in green and black tea.

Sample	B1 (*μ*g·kg^−1^)	B2 (*μ*g·kg^−1^)	G1 (*μ*g·kg^−1^)	G2 (*μ*g·kg^−1^)
Black tea (45)	Mean ± SD	Mean ± SD	Mean ± SD	Mean ± SD
	0.56 ± 0.99	0	1.46 ± 1.25	0
Green tea (15)	2.81 ± 0.007	0	0	0

**Table 4:** The frequency of fungal species contamination in black and green tea samples.

Fungi species	Black tea	Green tea	Total
*Aspergillus*	31 (66.7%)	9 (60%)	40 (65%)
*Mocur*	9 (20%)	7 (46.7%)	16 (26.7%)
*Penicillium*	16 (35.6)	7 (46.7%)	23 (38.3%)
*Fusarium*	0 (0.0%)	2 (13.3%)	2 (3.3%)
Yeast	3 (6.7%)	10 (66.7%)	13 (21.7%)
Black fungi	5 (11.1%)	2 (13.3%)	7 (11.7%)

**Table 5:** The frequency of samples contaminated with mycotoxins based on culture results.

Culture	AFT		OTA	
	>10 *μ*g·kg^−1^	≤10 *μ*g·kg^−1^	>10 *μ*g·kg^−1^	≤10 *μ*g·kg^−1^
Growth (55)	0	24	2	53
No growth (5)	0	0	1	4
Total (60)	0	24	3	57

## Data Availability

The excel file data used to support the findings of this study were supplied by Shiraz University of Medical Sciences under license and so cannot be made freely available. Requests for access to these data should be made to Keyvan Pakshir (pakshirk@gmail.com).
